# Adaptable Alchemy: Exploring the Flexibility of Specialized Metabolites to Environmental Perturbations Through Post-Translational Modifications (PTMs)

**DOI:** 10.3390/plants14030489

**Published:** 2025-02-06

**Authors:** Luca Cimmino, Annalisa Staiti, Domenico Carputo, Teresa Docimo, Vincenzo D’Amelia, Riccardo Aversano

**Affiliations:** 1 Department of Agricultural Sciences, University of Naples Federico II, Piazza Carlo di Borbone 1, 80055 Portici, Italyraversan@unina.it (R.A.); 2 Institute of Biosciences and Bioresources (CNR-IBBR), National Research Council of Italy, Via Università 133, 80055 Portici, Italy

**Keywords:** bioactive compounds, plant adaptation, (a)biotic stress, histone modification, metabolite–protein interactions

## Abstract

Plants are subjected to various stresses during the growth process, including biotic stresses, as well as abiotic stresses such as temperature, drought, salt, and heavy metals. To cope with these biotic and abiotic adversities, plants have evolved complex regulatory mechanisms during their long-term environmental adaptations. In a suddenly changing environment, protein modifiers target other proteins to induce post-translational modification (PTM) in order to maintain cell homeostasis and protein biological activity in plants. PTMs modulate the activity of enzymes and transcription factors in their respective metabolic pathways, enabling plants to produce essential compounds for their survival under stress conditions. Examples of post-translational mechanisms include phosphorylation, ubiquitination, glycosylation, acetylation, protein–protein interactions, and targeted protein degradation. Furthermore, the role of histone modifications in regulating secondary metabolism deserves attention due to its potential impact on heritability and its contribution to stress tolerance. Understanding the epigenetic aspect of these modifications can provide valuable insights into the mechanisms underlying stress response. In this context, also examining PTMs that impact the biosynthesis of secondary metabolites is meaningful. Secondary metabolites encompass a wide range of compounds such as flavonoids, alkaloids, and terpenoids. These secondary metabolites play a crucial role in plant defense against herbivores, pathogens, and oxidative stress. In this context, it is imperative to understand the contribution of secondary metabolism to plant tolerance to abiotic stresses and how this understanding can be leveraged to improve long-term survival. While many studies have focused on the transcriptional regulation of these metabolites, there is a growing interest in understanding various changes in PTMs, such as acetylation, glycosylation, and phosphorylation, that are able to modulate plants’ response to environmental conditions. In conclusion, a comprehensive exploration of post-translational mechanisms in secondary metabolism can enhance our understanding of plant responses to abiotic stress. This knowledge holds promise for future applications in genetic improvement and breeding strategies aimed at increasing plant resilience to environmental challenges.

## 1. Introduction

Plants are a plentiful source of different types of specialized bioactive metabolites. This wide chemical diversity, strongly favored by diverse natural selection, is the result of a complex biosynthetic machine and regulatory processes [1]. The type and amount of phytochemicals differ between and within species, tissues, and developmental stages. Metabolites are often produced during environmental stresses, working as osmoprotectants, antioxidants, and direct toxins against pests. However, they are also messengers, such as ligands for protein receptors or sensors, and serve as integral components in metabolism, acting as substrates, products, and cofactors of other metabolic pathways [2]. The cellular regulation of metabolite production during stress conditions involves all the levels of regulatory molecular biology, ranging from genetics to PTMs. This tangled network of plant stress response complicates the identification of metabolites that are effectively and functionally involved in tolerance to stress. In recent decades, significant progress has been achieved in understanding molecular regulation at the genetic and transcriptional levels (for a review, see Fernie and Tohge [3], Rai et al. [4], Fang et al. [5]). However, the present-day use of computational solutions as prediction tools and machine learning models have brought out hundreds of dynamics that affect plant response at a post-translational level [6]. PTMs increase the complexity of the proteome as response to environmental stresses. In plants, this is a growing field of research, and it has become cardinal in plant–environmental interaction studies. PTMs play critical roles in fine-tuning plant responses to stress, impacting cellular signaling, gene expression, protein stability and interactions, and enzyme kinetics [7]. Therefore, PTMs can design the metabolic repertoire of plants and can tailor the correct metabolic response to the environment, bestowing an additional advantage in the ecosystem.

With the aim of providing a starting point for research projects interested in elucidating PTMs in plants, in this review, we summarized recent advancements in the fields of the regulation of metabolites via PTM in response to abiotic stresses. In the first section, we underline the role of PTMs in modifying the stability, location, and function of target molecules, which is considered vital for survival in critical stress situations, enabling prompt response to a changing environment [8]. Then, we examine how PTMs influence the establishment of a transgenerational and non-transgenerational stress “memory”, working on the histone modifications, which are receiving increasing attention due to their critical role in mediating gene expression in response to environmental stressors [9]. Finally, we explored how protein–metabolite interactions, an area that remains largely underexplored, can serve as an additional layer of regulation beyond PTMs. Though this review focuses on a relatively novel field of research, we are confident that understanding how modifications of regulatory proteins affect metabolites will open up new scientific horizons. This knowledge could deepen our understanding of their role in plant physiology and pave the way for novel biotechnological approaches to enhance metabolic production.

## 2. The Impact of PTMs on Bioactive Metabolite Biosynthesis and Environmental Adaptation

Plant metabolites (phytochemicals) are commonly separated into primary and secondary metabolites and plant hormones, according to either their biochemical pathway or their function in plant biology. Primary metabolites are, for example, carbohydrates, lipids, and amino acids. These compounds are highly conserved and are directly involved in plant growth and development. Secondary metabolites (nowadays renamed as specialized metabolites) are regarded as the end products of primary metabolism, aiding plants in interacting with their biotic and abiotic environment. These include three major groups based on their core structure: polyphenols or phenolic compounds (including phenolic acids, flavonoids, and tannins), terpenoids (including carotenoids), and nitrogen/sulfur-containing compounds (as alkaloids and glucosinolates) [10,11]. Plant hormones (phytohormones) are small compounds that regulate organismal processes and growth. For plant hormones, there is still a debate about classifying them as primary (PM) or secondary metabolites (SM) [12,13]. PM and SM (including bioactive metabolites) are highly interconnected by smart molecular regulation. A multilayered regulation involving transcription factors and epigenetic modification can jointly or separately influence primary and secondary pathways in plants to protect them from stress stimuli [14]. Stressors for plants may arise from environmental conditions (abiotic stresses) or living organisms exhibiting pathogenic or parasitic characteristics (biotic stresses) [10,15]. In all these circumstances, PTMs can promptly elicit specialized metabolite production and even expand metabolome diversity, increase functionality, and allow for rapid responses, all at relatively low costs for the cell. As previously stated, PTMs have been studied in depth in various eukaryotic species; in plants, however, this area of interest is just emerging. Particularly, there is limited understanding of the direct in vivo effects of PTMs on metabolic enzymes or pathways, largely due to the absence of instruments capable of accurately measuring the input and output of these reactions [16]. At the core of the dynamics governing many PTMs lies the concept of reversibility. This process is facilitated by “writer” and “eraser” enzymes, which selectively target modified residues to either add or remove a specific modification [17]. In the following paragraphs, we describe each mechanism (such as phosphorylation, ubiquitination, and sumoylation), providing opportune examples of what is known regarding their involvement in metabolism, especially in response to abiotic stress.

### 2.1. Phosphorylation

Phosphorylation is the mechanism subjected to extensive, large-scale studies, particularly regarding its functional characterization [18]. It is defined as a covalent addition of the phosphate group on serine (Ser), threonine (Thr), and tyrosine (Tyr) residues [18]. This modification is catalyzed by kinases and reversed by phosphatases. Within plant genomes, there are more genes encoding kinases (940 in *Arabidopsis*) compared to phosphatases (150 in *Arabidopsis*), indicating that kinases may exhibit higher substrate specificity than phosphatases. Among hundreds of reported crop phosphatases and kinases, so far, only a small number have been studied experimentally. According to the reviewed literature, phosphorylation hotspots are over-represented in proteins with functions as effector proteins in signaling cascades, such as transcription or splicing factors. Typical examples are the proteins involved in light signaling transduction such as the PHYTOCHROME INTERACTING FACTORs (PIFs), transcriptional regulators belonging to the basic helix–loop–helix (bHLH) family. Among these, PIF3 (induced by light and auxin) harbors multiple phosphorylation sites. These increase negative charges around the activator motif, potentially altering the accessibility to transcriptional coactivators [19]. Regarding the direct effects of protein phosphorylation affecting metabolite production, several studies demonstrated the role of phosphorylation in response to a changing environment, altering both primary and specialized metabolites [20,21]. For example, Hu et al. [22] examined the phosphoproteome and metabolic changes in maize leaves subjected to heat, drought, and combined stress by using multiplex iTRAQ-based quantitative proteomic and LC-MS/MS methods. At a metabolic level, the authors found that the three analyzed stresses increase the phosphorylation levels of fructose-bisphosphate aldolase, a key enzyme in the gluconeogenesis pathway, decreasing protein expression. Similar results were also found in tomato (*Solanum lycopersicum* L.), when in response to drought stress, this gene was downregulated [23]. Since gluconeogenesis consumes a high quantity of energy, the inhibition of gluconeogenesis seems to directly affect energy management and the consequent plant adaptation to abiotic stresses. The role of protein phosphorylation in regulating primary metabolism was also demonstrated in tomato during postharvest cold storage [24]. The authors observed a significant alteration in the phosphorylation status of many enzymes involved in primary metabolism, such as fatty acid desaturase, sucrose synthase, and alcohol dehydrogenase. This suggested that during low-temperature storage, the alteration of enzymes involved in primary metabolism affects ROS scavenging and metabolism, which is fundamental for cell survival. Regarding specialized metabolites, for example, it was shown that specific phosphorylation events by MAP kinase 4 (MPK4) in *Arabidopsis* are necessary for the activation of genes related to anthocyanin production [25]. Given that MPK4 is recognized for its significant roles in plant immunity and jasmonic acid signaling [26], and to be activated by abiotic stress such as cold and osmotic stress [27,28], MPK4 likely contributes to anthocyanin accumulation during these stimuli. Recent studies have highlighted that the microbiome also can modulate plant tolerance to abiotic stresses through post-translational modifications (PTMs). For instance, specific modifications, such as acetylation and phosphorylation, regulate microbe–plant communication and metabolic adaptation under environmental stress conditions [29]

### 2.2. Glycosylation

Glycosylation is acknowledged as one of the most complex PTMs due to the diverse structures of glycans and the multitude of glycosylation sites on the peptide backbone. In eukaryotic cells, glycosylation primarily occurs as N-glycosylation or O-glycosylation, depending on the site of attachment on proteins, within the endoplasmic reticulum and Golgi apparatus [30]. N-glycosylation involves attaching an oligosaccharide to the amino acid asparagine (Asn) within a specific sequence motif, Asn-X-Ser/Thr-X, where X represents any amino acid except proline (Pro). O-glycosylation takes place on the hydroxyl groups of serine (Ser) and hydroxyproline (Hyp) residues within specific amino acid motifs, primarily in the structural proteins belonging to the hydroxyproline-rich glycoprotein (HRGP) families [31,32]. Plants possess distinct forms of O-glycosylation that differ from those found in animals and prokaryotes, playing a crucial role in modulating functions of secretory and nucleocytoplasmic proteins, such as regulating transcription, protein localization, and degradation. O-glycosylation has been shown to influence development and environmental acclimatization [33]. Mutations at the early stages of N-glycan assembly result in severe plant growth phenotypes [32] and may be lethal due to an accumulation of unfolded proteins in the endoplasmic reticulum. To cope with abiotic stress, glycosylation plays a role in controlling biological processes and enhancing their adaptability. For example, in tomato, the protein SlC3H39, a zinc-finger protein containing a CCCH domain, plays a role in the response to cold stress. Under cold stress conditions, SlC3H39 localizes stress granules (SGs) and processing bodies (PBs), cellular structures involved in stress responses, mRNA stability, and translation regulation. The enzyme SlSEC1, an O-GlcNAc transferase, catalyzes the O-GlcNAcylation of SlC3H39, which involves adding an N-acetylglucosamine (GlcNAc) group to the protein. This modification enhances stability and preserves the function of the protein. This modification is crucial for maintaining the protein’s functionality under cold conditions, allowing the plant to better cope with thermal stress [33].

### 2.3. Ubiquitination and SUMOylation

Ubiquitination serves as a versatile PTM that mediates growth, development, and stress response in all eukaryotic species. Ubiquitin, a compact polypeptide consisting of 76 amino acids, is known for its stability and high conservation across species. It is commonly attached to a Lysine residue in target proteins through a series of enzymatic actions involving three key enzymes: E1 (an ATP-dependent ubiquitin-activating enzyme), E2 (a ubiquitin-conjugating enzyme), and E3 (a ubiquitin protein ligase) [34]. In plants, several large-scale proteomic studies of ubiquitination have been undertaken on Arabidopsis [35], rice (*Oryza sativa*) [36], and soybean (*G. max*) [37]. These studies have revealed that polyubiquitination frequently leads to targeted degradation of transcription factors and components of signaling pathways (e.g., receptors and kinases), resulting in many downstream effects, including metabolic alteration, such as photosynthesis, carbohydrate, energy metabolism, and specialized metabolism during environmental stress. For example, in Arabidopsis, the CONSTITUTIVELY PHOTOMORPHOGENIC 1 (COP1)/SUPPRESSOR OF PHYA-105 (SPA) ubiquitin ligase complex regulates anthocyanin accumulation under low-light conditions. Mutations in COP1/SPA complexes result in a hyperaccumulation of anthocyanins under both normal and excess light conditions, highlighting the importance of normal light signaling via COP1/SPA in preventing excessive anthocyanin accumulation under high-light conditions [38]. In tea (*Camellia sinensis* L.), global ubiquitome profiling under drought stress revealed a large number of ubiquitinated proteins participating in metabolic pathways, including ubiquitin-mediated proteolysis, catechins biosynthesis, and carbohydrate and amino acid metabolism [39]. This suggests that ubiquitination of key metabolic enzymes plays an important role in drought adaptation. In addition to ubiquitins, plants utilize SUMOylation, a related PTM where a small ubiquitin-like modifier (SUMO) is covalently attached to substrate proteins [40]. Unlike ubiquitin, SUMO is usually not associated with protein degradation but can interfere with protein activity, location, and half-life and modulate protein–protein interactions [41]. In plants, SUMOylated proteins rapidly and robustly accumulate in response to oxidative, drought, salt, and temperature stresses and abscisic acid treatment [42]. While direct ubiquitination or SUMOylation of metabolites remains poorly understood, recent research has underscored the significance of ubiquitination in phytohormone signaling pathways. Thus, these modifications regulate key hormones involved in stress response, including abscisic acid, auxin, brassinosteroid, cytokinin, ethylene, jasmonic acid [43].

### 2.4. PTMs’ Crosstalk

Many types of PTMs often occur simultaneously and crosstalk, which contributes to the proteomic and metabolomic response of plants subjected to environmental stressors. Emerging evidence suggests that modifications at one site can be dependent on modifications occurring on neighboring residues. Certainly, evidence indicates crosstalk between SUMOylation and phosphorylation in processes related to signaling, nutrient acquisition, and plant growth [44]. The first phosphorylation–sumoylation crosstalk in plants was identified in the basic helix–loop–helix (bHLH) transcription factor CESTA (CES), a component of the brassinosteroid response machinery [45]. Following treatment with brassinosteroid, CES undergoes robust SUMOylation mediated by SUMO1 and SUMO2 at lysine 72 (K72), leading to its relocation to nuclear bodies. This serves as a prime illustration of a system in which crosstalk among various PTMs within a single protein initiates sequential signaling cascades, spanning from activation to turnover, thereby ensuring a dynamic yet tightly controlled regulation that is a primary key for plant adaptation during stress conditions.

## 3. Epigenetic Regulation via Histone Modifications and Its Role in Metabolite Adaptation to Environmental Stress

Epigenetics focuses on heritable changes in gene expression that occur without altering the underlying DNA and RNA sequences [46]. Epigenetic mechanisms encompass nucleosome positioning, DNA methylation, histone variants and modifications, and small RNAs, collectively defining the epigenome [47]. Among those involved in PTMs, histone modifications warrant specific attention, as they involve chemical changes to histone proteins that play a key role in DNA packaging and gene expression regulation. However, their role in this context remains largely unexplored. Histone modifications involve chemical changes to the N-terminal tails of the histone proteins that protrude from the nucleosome core, including acetylation, methylation, phosphorylation, ubiquitination, and glycosylation. These modifications establish specific epigenetic states that enable the genome to adapt to environmental changes [48,49]. However, while certain aspects of histone modifications, such as methylation/demethylation and acetylation/deacetylation, have been studied, particularly for their influence on transcription by controlling the accessibility of transcription factors to DNA [50,51], their broader role in the epigenetic regulation of plant stress responses remains largely unexplored. A review by Liu et al. [52] provides a comprehensive overview of the dynamics of DNA methylation and histone modifications in plant responses to abiotic stresses, offering insights into the epigenetic mechanisms involved.

### 3.1. Histone Methylation and Demethylation

Histone methylation and demethylation can either repress or activate gene expression, depending on the modification site. Methylation of lysine 4 on histone 3 (H3K4me3) and methylation of lysine 36 on histone 3 (H3K36) promote transcription by opening chromatin, whereas methylation at lysine 9 (H3K9me2) and lysine 27 (H3K27me3) compacts chromatin and represses transcription [53]. Conversely, demethylation at these sites has opposite effects: removing methyl groups from H3K4me3 typically represses, while demethylating H3K9me2 or H3K27me3 activates gene expression by eliminating repressive methylation marks [54]. These modifications are crucial for regulating developmental genes and for influencing metabolite production in response to environmental stresses. For example, light exposure in apple (*Malus domestica*) leads to demethylation of histone H3K27me3, enhancing transcription of genes like *MdMYB10* and *MdMYB1* and promoting anthocyanin production [55]. Similarly, the *JMJ25* gene, a histone H3K9 demethylase from *Populus trichocarpa*, is known to suppress anthocyanin biosynthesis. Its expression, induced under dark conditions, suggests a stress-response mechanism where plants conserve energy by reducing secondary metabolites like anthocyanins. ChIP-qPCR assays confirmed the role of *JMJ25* in dynamically demethylating H3K9me2 at the MYB182 chromatin [56]. In *A. thaliana*, *JMJ15*, which encodes an H3K4 demethylase, enhances salt tolerance. Overexpression of this gene results in shortened plants and increased lignin in stems, which contributes to their salt resistance [57,58]. Recently, research has increasingly focused on the divergence of chromomethylases and their role in plant adaptation to environmental stresses. For example, the divergence between CMT2 and CMT3, two chromomethylases, has allowed them to specialize in distinct functions, contributing to DNA methylation regulation. Jiang et al. [59] demonstrated that CMT2 interacts with the modified histone H3K9me to guide CHH methylation in heterochromatic regions, contributing to transposon silencing and genome stability. Additionally, CMT2 has an N-terminal region that regulates its stability under thermal stress, suggesting a possible role of these epigenetic mechanisms in plant responses to challenging environmental conditions.

### 3.2. Histone Acetylation

**Figure 1:**
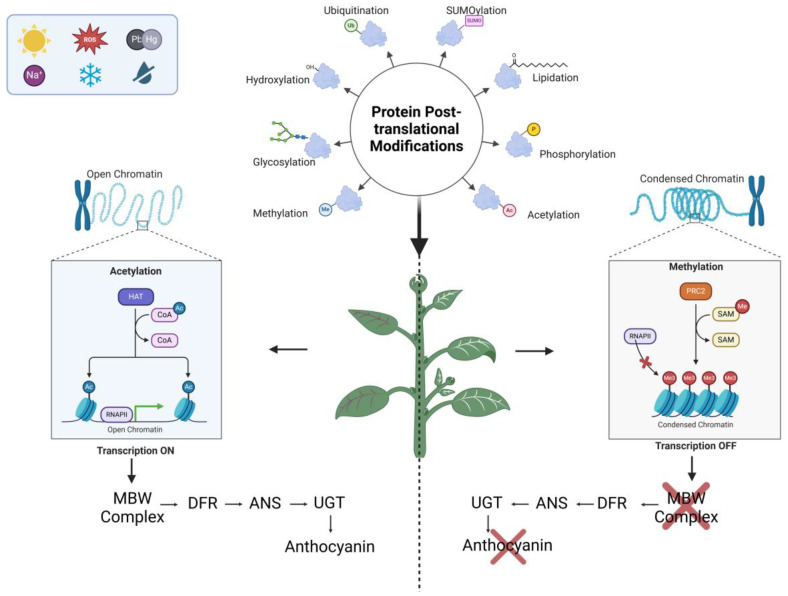
Abiotic factors trigger post-translational modifications (PTMs) in plants, influencing chromatin structure and gene expression. An example of anthocyanin biosynthesis is presented: on the left, acetylation (Ac) opens chromatin, allowing transcriptional activation of the MBW complex and downstream enzymes (*DFR*, *ANS*, *UGT*), leading to anthocyanin production. On the right, methylation (Me) condenses chromatin, inhibiting transcription of the same biosynthetic pathway, preventing anthocyanin accumulation. This highlights how PTMs, such as acetylation and methylation, regulate chromatin dynamics in response to environmental stimuli. Created in BioRender: https://BioRender.com/k98j551 (accessed on 2 December 2024).

Histone acetylation, such as that occurring at lysine residues H3K9 and H3K27, generally enhances gene expression by making chromatin more open and accessible to transcription factors. For example, salt stress induces acetylation of histone H3K9 in the promoter region of stress-responsive genes, contributing to their activation in plants like maize (*Zea mays*) and black cottonwood (*P. trichocarpa*) [60,61]. In salt-stressed maize, plant response is accompanied by increased levels of global histone H3 acetylation at K9, which has been shown to control the increase in cell wall proteins such as expansins and the plasma membrane proton pump [60]. In contrast, deacetylation typically represses gene expression, highlighting a dynamic regulation of chromatin states in response to environmental conditions. Further studies have highlighted the role of histone acetylation in response to other stresses. For example, drought stress leads to a reduction in H3K9ac, which is associated with the downregulation of genes involved in the synthesis of flavonoids and abscisic acid (ABA), both of which are crucial for plant stress responses. Treatment with the histone deacetylase inhibitor trichostatin A (TSA) attenuated these effects, enhancing gene expression linked to flavonoid and ABA synthesis, thus improving drought tolerance [62]. Additionally, UV-B irradiation increases H3K9 acetylation at flavonol metabolic pathway genes, enhancing their activity (Figure 1). This response can be modulated by treatment with the PAMP flg22 peptide, a bacterial immune response elicitor, which suppresses H3K9 acetylation and represses flavonol gene expression. Similar regulatory patterns have been observed in hemp (*Cannabis sativa*), where expression levels of Histone Acetyltransferase (HAT) genes significantly change in response to various abiotic stresses (e.g., cold and salt), impacting the synthesis of cannabinoids. Suppression of these HATs through treatment with the inhibitor PU139 has been shown to negatively impact cannabinoid production, highlighting the critical role of histone acetylation in managing plant responses to environmental stress [63]. A recent study by Yu et al. [64] explores the role of lysine acetylation of the histone acetyltransferase adaptor protein ADA2 as a metabolic control mechanism that connects cellular acetyl-CoA levels to chromatin modifications in plants. Acetyl-CoA, a key metabolite in cellular metabolism, acts as a donor for acetyl groups in histone acetylation reactions. Research reveals that specific lysine residues on ADA2 are acetylated in response to fluctuations in acetyl-CoA levels, acting as a “sensor” to regulate histone acetyltransferase (HAT) activity. When acetyl-CoA levels rise, ADA2 acetylation increases, boosting HAT activity and histone acetylation, which opens chromatin and activates stress-responsive genes. Conversely, lower acetyl-CoA levels reduce ADA2 acetylation, restricting HAT activity and chromatin accessibility.

### 3.3. Histone Variants and Less Well-Known PTMs

Recent studies have revealed an additional layer of complexity: histone variants such as H2A.Z can interact with histone modifications to further modulate gene activity. H2A.Z is a variant of the histone H2A, evolutionarily conserved, that integrates into nucleosomes and plays key roles in gene regulation and chromatin stability. The H2A.Z variants differ from canonical H2A at many positions throughout the primary sequence, with the main differences observed in the carboxy-terminal α-helix included in the docking domain. Specifically, in wild-type Arabidopsis plants SW1, the expression of anthocyanin biosynthesis genes is inhibited through the incorporation of H2A.Z into the nucleosomes of these genes. This incorporation hinders the accumulation of H3K4me3, a mark associated with active transcription, and ultimately suppresses the expression of anthocyanin biosynthesis genes [65]. Similarly, in mutants with defects in H2A.Z deposition (arp6/pie1/hta9 hta11), an elevation in H3K4me3 levels is observed. This increase is linked to enhanced expression of anthocyanin biosynthesis genes, causing accumulation of such pigments in these mutants [65]. These findings underscore the intricate interplay between histone variants and modifications in regulating key metabolic pathways, suggesting that answers to these and related questions may clarify the mechanisms underlying metabolic regulation in plants. Other less well-known histone modifications involve the covalent addition of molecules, such as phosphate groups, ubiquitin, and SUMO, which can influence gene expression and stress responses depending on the histone modifications site. For example, phosphorylation of histone H3 at Serine 10 (H3S10) is associated with transcriptional activation, while phosphorylation at Serine 9 (H3S9) correlates with repression. Ubiquitination of histone lysine residues can act as a marker for both activation (e.g., H2Bub with H3K4me3) and repression (e.g., H2Aub with H3K27me3). In upland cotton (*Gossypium hirsutum*), ubiquitination has also been linked to the regulation of organ size [66]. SUMOylation, in contrast, plays a role in responses to environmental stresses, as shown by the increased SUMO signals associated with chromatin during heat stress [67]. Small RNAs (such as microRNAs, siRNAs, and piRNAs) also play a crucial role in modulating post-translational modifications (PTMs). These RNAs not only act as intermediaries in gene expression regulation but also closely interact with proteins involved in RNA methylation, such as METTL3, METTL14, and ALKBH5, serving as “readers”, “writers”, and “erasers”. For instance, these proteins are subjected to phosphorylation and ubiquitination influencing their catalytic activity, stability, and subcellular localization and, consequently, the modifications on methylated RNAs. Small RNAs can guide these proteins toward specific molecular targets, establishing dynamic circuits of epigenetic regulation. For example, it has been shown that phosphorylation of METTL3 promotes the formation of the METTL3/METTL14/WTAP complex, thereby increasing m6A methylation levels in specific transcripts, with significant impacts on processes such as stem cell differentiation and DNA damage response [68].

Despite advancements in understanding these mechanisms, the role of these PTMs in metabolite biosynthesis under stress remains unclear, highlighting the need for further studies to elucidate their involvement in transcriptional control during plant adaptation to abiotic stresses. It would be interesting to further investigate how these modifications contribute to long-term gene expression changes in response to various environmental stresses. Understanding this could clarify how plants utilize epigenetic mechanisms for adaptive resilience.

### 3.4. Histone Modifications Serve as a Memory for Metabolic Adaptation, Even Across Generations

The retention of molecular experiences, often referred to as “stress priming”, allows plants to recall past stress episodes and utilize the stored information for more efficient adaptation to new conditions [69,70]. The term “priming” has gained prominence, signifying that mild exposure to stress can activate plant stress responses, enabling faster and stronger reactions upon subsequent stress occurrences. This adaptive mechanism involves the establishment of an epigenetic memory that plays a crucial role in helping plants overcome future stress challenges [69,71,72]. The duration of memory varies depending on the type of environmental exposure. Cold memory induced by vernalization can last until reproductive growth, while most stress-induced memories will be short-term if the stress does not recur. These signals not only modulate plant response to the surrounding environment but can also be maintained during plant development or leave an inheritable epigenetic imprint across generations. Indeed, memory can be divided into two main categories: somatic memory and transgenerational memory. The former involves epigenetic changes in an organism that occur in response to environmental stress. Evidence shows that PTMs play a key role in regulating somatic stress memory, particularly in relation to primary and secondary metabolism. For example, in meadow foxtail (*Alopecurus pratensis*), plants subjected to repeated waterlogging and drought stress demonstrated enhanced recovery. Indeed, they displayed higher levels of Rubisco content, antioxidative enzymes (POX, SOD), and chlorophyll b, all indicators of long-term drought stress memory [73]. These responses are tightly regulated by histone modifications. Under cold stress, Arabidopsis plants showed reduced H3K27me3 deposition at specific cold-responsive genes such as *COR15A* and *GALACTINOL SYNTHASE 3* (*GOLS3*), which regulate the biosynthesis of raffinose oligosaccharides (RFOs). This reduction persisted even after the stress, suggesting that H3K27me3 may function as an epigenetic marker for stress memory [74]. Similarly, drought memory in rice was associated with increased H3K4me3 levels observed at key genes involved in proline biosynthesis, crucial for stress tolerance [69]. This indicates that histone modification is involved in plants’ ability to “remember” and respond more effectively to future drought events during their life cycle. Salt stress also induces changes in the distribution of H3K27me3 marks, influencing somatic stress memory. This alteration allows the release of previously repressed genes, such as HKT1, enabling them to respond more effectively and rapidly to future salt stress episodes [75]. Additionally, the maintenance of elevated levels of H3K4me3 at the locus of Δ1-pyrroline-5-carboxylate synthetase 1 (P5CS1), a key enzyme involved in proline synthesis during recovery after salt stress, further reinforces the role of histone modifications in establishing salt stress memory and ensuring better recovery from such conditions [76]. Transgenerational memory, on the other hand, involves the transmission of epigenetic modifications across generations. It is a less well-studied and more elusive phenomenon compared to somatic memory. However, its comprehension and interpretation are less explored. Lukić et al. [77] demonstrated that maternal stress experiences, such as drought or waterlogging, can precondition offspring to respond better to similar stress, with higher biomass and phenolic compounds production. This suggests a possible link between epigenetic marks, including PTMs, and metabolite regulation in stress response. Although research shows that histone modifications can influence secondary metabolite biosynthesis under stress (Table 1), it remains uncertain whether this regulation forms a memory of prior biosynthetic states. Further investigation into how PTMs store information about metabolite production could unlock opportunities to enhance the production of important compounds in agriculture and medicine.

## 4. Metabolites as PTM Effectors

**Figure 2:**
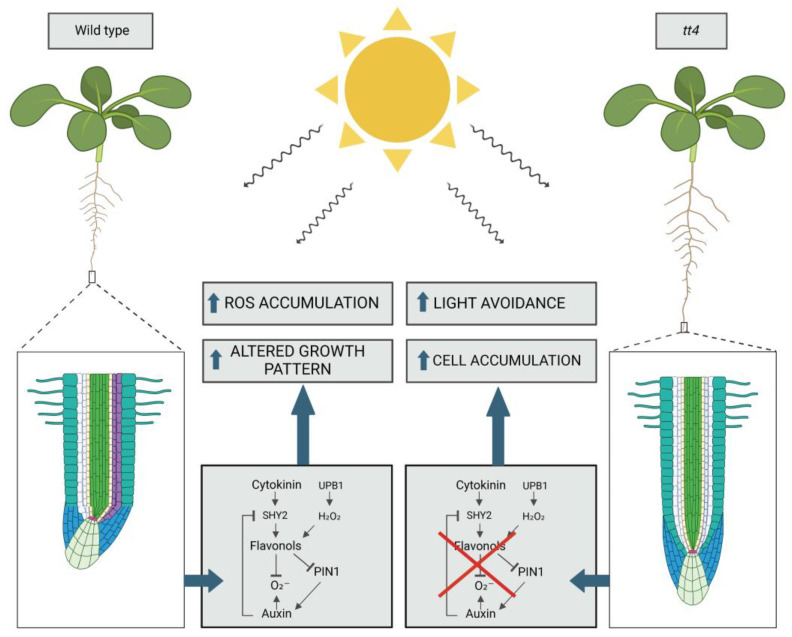
Metabolites as post-translational modification (PTM) effectors in root growth regulation. In wild-type plants, flavanols and anthocyanins regulate the balance between cell division and differentiation in root meristems. Light induces asymmetric accumulation of flavonols and anthocyanins on the exposed side (depicted in purple in the image on the left), promoting cell differentiation and limiting proliferation. This results in light avoidance by the roots, leading to reduced growth and a shift in the meristem away from the light source. Flavonols inhibit auxin polar transport mediated by PIN1 and scavenge the superoxide anion (O^2−^), contributing to the regulation of the boundary between the proliferative and differentiation zones. In the *tt4* mutant, which does not produce anthocyanins, the light response is impaired. In the absence of anthocyanins, the roots do not asymmetrically accumulate flavonols and do not exhibit light avoidance. Consequently, the root growth in the *tt4* mutant is greater compared to the wild type, with a regular zonation between cell proliferation and differentiation, as the roots do not limit growth in response to light. This highlights the importance of anthocyanins and flavonols inducing altered growth responses under light conditions. Created in BioRender: https://BioRender.com/p40c968 (accessed on 2 December 2024).

In genetics and molecular biology courses, the regulation of the lac operon in *Escherichia coli* is often emphasized as a classic example of gene expression regulation. While this example does not involve canonical post-translational modifications (PTMs), such as glycosylation or phosphorylation, the interaction between allolactose (a metabolite) and the repressor protein LacI induces conformational changes that alter its functionality. This regulatory mechanism, though similar to allosteric regulation, also represents an additional layer of post-translational regulation. Specifically, metabolite–protein interactions, like those between allolactose and LacI, influence protein function in a non-covalent manner, thereby expanding the concept of PTMs. This metabolite-induced regulation modulates the downstream activity of enzymes involved in lactose metabolism, enabling the microorganism to adapt to environmental changes. These types of protein–metabolite interactions can therefore be seen as an additional step in the broader context of post-translational regulation, extending the impact of PTMs across various cellular processes. Over the last decade, evidence has increasingly shown that specialized metabolites, alongside primary metabolites *sensu lato,* may act as regulators of plant growth and defense by interacting commonly with protein partners, including those from host–microbe interactions [79]. These interactions can also be modulated according to changes in protein and metabolite concentrations and their post-translational status, impacting cellular function and prevent toxicity [80]. Distinct metabolites, such as terpenes, phenolics, and glucosinolates, demonstrate functions similar to those of plant hormones. For example, studies on *Arabidopsis* tt4 (*transparent testa4*) mutants defecting in flavonoid accumulation have shown that flavonoids modulate auxin transport by interacting with auxin transporters and transport-regulating proteins like ABCBs and PIN-FORMED (PIN), leading to altered growth patterns [81]. Additionally, *tt4* mutants exhibited reduced light avoidance, linked to reduced auxin polar transport and reduced ROS accumulation, which influence cell division and elongation [82] (Figure 2). Flavonols may also act as antioxidants, influencing the cellular redox state and signaling pathways by modifying protein structures [83]. Other secondary metabolites such as 3-hydroxypropyl-glucosinolate regulate development by interacting with key regulatory protein the Target of Rapamycin (TOR) kinase, which is crucial for growth regulation [84]. The evolutionary newer nature of glucosinolate [85] suggests that plants might utilize specialized metabolites as a flexible repertoire to enhance their ability to thrive in diverse and challenging environments.

## 5. Conclusions

While technological advancements have significantly enhanced our understanding of specialized metabolites, comprehensively grasping the myriad interactions and functionalities of these compounds remains a complex and unresolved issue. The role of lesser-known PTMs, including histone variants, in regulating metabolite biosynthesis under stress is still unclear, highlighting the need for further research on their impact on transcriptional control during stress adaptation. The emerging understanding of the extensive roles of post-translational modifications (PTMs) offers a unique perspective on the dynamic regulation of biological systems. PTMs introduce an additional regulatory layer that fine-tunes protein function after translation. Further, PTMs exemplify how feedback loops and bidirectional interactions shape cellular processes, demonstrating that regulatory mechanisms extend beyond genetic coding with an impact on the phenotype. Recognizing these complexities not only enhances our understanding of plant stress responses but also prompts a profound reconsideration of our view of biological process. Additionally, it remains uncertain whether histone modifications can establish a memory of past biosynthetic states, which could lead to new opportunities for improving agricultural and medicinal compounds. Understanding protein–metabolite interactions is also crucial, as they may serve as multifunctional components in stress response. Initial findings suggest that these interactions are key to environmental adaptation, offering potential for enhancing stress resilience in crops. However, this field is still developing, and standardized methods for integrating high-throughput data are needed. Multi-omics approaches, combining metabolomics, epigenomics, and interactomics, offer additional promising pathways to unravel these complexities. While our knowledge on how secondary metabolites are regulated by PTMs remains limited, further investigation could reveal new strategies for developing stress-tolerant plants. Addressing questions about decoding the hidden functions of metabolites, and predicting their modifications and their heritability, holds promises to unlock new perspectives on biological regulatory processes.

## Figures and Tables

**Table 1:** Summary of histone modifications and PTMs in different plant species and their influence on secondary metabolite production under abiotic stress conditions.

Histone Modification	Plant Species	Secondary Metabolites	Histone Mark	Abiotic Stressor	References
Demethylation	*Arabidopsis thaliana*	Lignin	H3K4me3	Salt	[58]
Acetylation	*Arabidopsis thaliana*	Flavonol	H3K9ac	UV-B radiation	[78]
Demethylation	*Poplar* (*Populus* spp.)	Anthocyanin	H3K9me2	Dark treatment	[56]
Methylation	*Arabidopsis thaliana*	Anthocyanin	H3K4me3	-	[65]
Acetylation	*Cannabis sativa*	Cannabinoid	H3K9ac	Cold and salt stress	[63]
Deacetylation	*Hippophae rhamnoides*	Flavonoids and abscisic acid (ABA)	H3K9ac deacetylated	Drought stress	[62]

## Data Availability

The original contributions presented in the study are included in the article, further inquiries can be directed to the corresponding author.
